# Recuperación de bacterias aerobias y anaerobias de pacientes con apendicitis aguda mediante botellas de hemocultivo

**DOI:** 10.7705/biomedica.4774

**Published:** 2019-12-30

**Authors:** Adriana Jiménez, Andrés Sánchez, Andrés Rey, Claudia Fajardo

**Affiliations:** 1 Unidad de Prevención y Control de Infecciones, Hospital de San José, Bogotá, D.C., Colombia Unidad de Prevención y Control de Infecciones Hospital de San José BogotáD.C Colombia; 2 Facultad de Medicina, Fundación Universitaria de Ciencias de la Salud, Bogotá, D.C., Colombia Facultad de Medicina Fundación Universitaria de Ciencias de la Salud BogotáD.C Colombia; 3 Departamento de Cirugía, Hospital de San José, Bogotá, D.C., Colombia Departamento de Cirugía Hospital de San José BogotáD.C Colombia

**Keywords:** bacterias anaerobias, bacterias aerobias, apendicitis, apendicectomía, líquido ascítico, pruebas de sensibilidad microbiana, Bacteroides fragilis, Bacterias, anaerobic, bacteria, aerobic, appendicitis, appendectomy, ascitic fluid, microbial sensitivity tests, Bacteroides fragilis

## Abstract

**Introducción.:**

La apendicitis aguda es la primera causa de abdomen agudo; sin embargo, poco se conoce sobre las bacterias asociadas y su perfil de sensibilidad.

**Objetivo.:**

Identificar y determinar el patrón de resistencia de las bacterias aerobias y anaerobias aisladas en cultivo de líquido periapendicular tomado de los pacientes con apendicitis aguda, y establecer la proporción de bacterias según la fase clínica.

**Materiales y métodos.:**

Se llevó a cabo un estudio descriptivo y prospectivo en el Hospital Universitario de San José de Bogotá (Colombia), en pacientes mayores de 16 años sometidos a apendicectomía abierta. Se tomaron muestras de líquido periapendicular, las cuales se sembraron directamente en botellas de hemocultivos para aerobios y anaerobios.

**Resultados.:**

Se incluyeron 154 pacientes. Del total de cultivos, el 87 % (n=134) fueron positivos: 77 % (n=118) para aerobios y 51 % (n=79) para anaerobios. La proporción de cultivos positivos fue inferior en los casos de apendicitis no complicada, en comparación con aquellos de apendicitis complicada (80 % (66/83) Vs. 95 % (67/71); p=0,003). Los microorganismos aislados con mayor frecuencia fueron: *Escherichia coli* (53 %) (n=84), *Bacteroides* sp. (25 %) (n=25), *Propionibacterium acnes* (21 %) (n=21), *Staphylococci* coagulasa negativo (17 %) (n=27), *Enterococcus* sp. (10 %) (n=15) y Fusobacterium sp. (11 %) (n=11). La sensibilidad de *E. coli* a la amplicilina sulbactam fue de 30 %. La sensibilidad de Bacteroides spp. a la clindamicina y la ampicilina sulbactam fue de 91 %. El 100 % de los anaerobios fueron sensibles a piperacilina tazobactam, ertapenem, meropenem y metronidazol.

**Conclusiones.:**

Los cultivos intraoperatorios son pertinentes en la apendicitis para determinar el patrón epidemiológico local, y establecer los antibióticos profilácticos y terapéuticos para esta enfermedad. Su siembra directa en botellas de hemocultivo permite una gran recuperación de microorganismos.

## Abstract (en)

**Introduction:**

Acute appendicitis is the first cause of acute abdomen, however, there is a little information about the associated bacteria and its sensibility profile.

**Objetive:**

To identify and to determine the resistance pattern of aerobic and anaerobic bacteria isolated in periapendicular fluid cultures taken in patients with acute appendicitis and to establish the proportions of isolates according to the clinical phase.

**Materials and methods:**

A descriptive and prospective study was undertaken at the Hospital Universitario de San José (Bogotá, Colombia) of patients older than sixteen years of age, undergoing an open appendectomy. A sample of periappendiceal fluid was taken, which was deposited directly into aerobic and anaerobic blood culture bottles.

**Results:**

One hundred and fifty-four patients were included. The overall positivity of cultures was 87% (n=1344); 77% (n=118) for aerobes and 51% (n=79) for anaerobes. The proportion of positive cultures was lower in the uncomplicated appendicitis cases as compared to the complicated ones (80% (66/83) vs. 95%(67/71), p = 0.003). The microorganisms isolated most frequently were: *Escherichia coli* (53%) (n=84); *Bacteroides* spp. (25%) (n=25); *Propionibacterium acnes* (21%) (n=21); *coagulase negative Staphylococci* (17%) (n=27); *Enterococcus* spp. (11%) (n=15), and *Fusobacterium* spp. (11%) (n=11). The sensitivity of E.coli to ampicillin/sulbactam was 30%. The sensitivity of *Bacteroides* spp. to clindamycin and ampicillin/sulbactam was 91%. All anaerobe isolates were sensitive to piperacillin/tazobactam, ertapenem, meropenem and metronidazole.

**Conclusions:**

Intraoperative cultures in acute appendicits are relevant in order to determine the local epidemiological pattern and to establish prophylactic and therapeutic antibiotics for this pathology; direct inoculation in blood culture bottles allows a high recovery of microorganisms.

La apendicitis aguda es la primera causa de abdomen agudo en nuestro país y en el mundo, con una incidencia estimada de 7 a 14 % a lo largo de la vida ^1^.

El manejo integral de los pacientes con apendicitis aguda requiere del conocimiento de las variables clínicas, de la técnica quirúrgica y de los agentes patógenos asociados con sus correspondientes perfiles de sensibilidad antibiótica, para optimizar el manejo antibiótico profiláctico y terapéutico, e incluso, para considerar la opción no quirúrgica.

La efectividad de la profilaxis antibiótica fue demostrada por un estudio danés de los 80, en el cual se estableció la incidencia de infección del sitio operatorio en la apendicitis aguda en fase inflamatoria, con profilaxis y sin ella en 1,9 y 7 %, y en apendicitis gangrenosa en 8,3 y 30,6 %, respectivamente ^2^. Para la elección apropiada del antibiótico profiláctico y el terapéutico, se deben conocer los microorganismos implicados y su patrón de sensibilidad; sin embargo, en las últimas décadas, decayó la toma rutinaria de cultivos en los casos de apendicitis debido a la presunción de que se aislarían los microorganismos usuales. Actualmente, debido al aumento de la resistencia a los antibióticos, en particular, al aumento de la resistencia a la ampicilina sulbactam en la enterobacterias ^3^ y a la clindamicina ^4^ en Bacteroides spp., esta práctica ha recobrado su pertinencia.

En Latinoamérica, Colombia incluida, se han llevado a cabo algunos estudios sobre los agentes etiológicos asociados a las infecciones intrabdominales, pero no específicamente en los casos de apendicitis, ni se han establecido los agentes patógenos anaerobios implicados y su sensibilidad ^5^^,^^6^.

El cultivo de las bacterias anaerobias constituye un reto microbiológico pues estas pierden rápidamente su viabilidad por errores en la fase preanalítica y, si bien los equipos automatizados pueden identificarlas, no pueden ejecutar las pruebas de sensibilidad, las cuales deben hacerse necesariamente con un método manual.

Debido a dicho vacío del conocimiento, se diseñó este estudio con el propósito de identificar y determinar el patrón de resistencia de las bacterias aerobias y anaerobias aisladas en los cultivos de líquido periapendicular de aquellos pacientes con apendicitis aguda y, además, establecer la relación con la fase clínica, con el fin de brindar el sustento teórico para la creación de protocolos regionales de uso de antibióticos en la infección intraabdominal.

## Materiales y métodos

Se trata de un estudio observacional, descriptivo y prospectivo, llevado a cabo entre junio de 2014 y enero de 2015 en el Hospital de San José (Bogotá, Colombia), una institución universitaria de atención privada con 300 camas y, aproximadamente, 15.000 egresos al año.

Se incluyeron los pacientes mayores de 16 años con diagnóstico de apendicitis y que fueron sometidos a apendicectomía abierta. Se extrajeron las variables demográficas y clínicas de la historia clínica (fase clínica de la apendicitis de acuerdo con los hallazgos macroscópicos en el periodo intraoperatorio, el tipo y la duración del tratamiento antibiótico, las complicaciones posoperatorias a los 30 días y la clasificación definitiva de la apendictis según el diagnóstico histopatológico).

### Métodos de microbiología

De cada paciente se obtuvo una muestra de 10 ml de líquido periapendicular según las recomendaciones de la *Infectious Diseases Society of America*^7^. Inmediatamente y en las salas de cirugía, la muestra se sembró en botellas de hemocultivos para aerobios y anaerobios, las cuales se procesaron en un equipo automatizado BacT/ALERT 3D™ (bioMérieux).

Los cultivos positivos se recuperaron en agar sangre, MacConkey o agar Brucella con 5 % de sangre de cordero, enriquecido con hemina y vitamina K_1_ para anaerobios.

La identificación de los microorganismos aislados, aerobios y anaerobios, se realizó con el equipo MicroScan™ (Beckman Coulter). Las pruebas de sensibilidad para aerobios se ejecutaron con el mismo equipo automatizado y, para anaerobios, se empleó la prueba de la tira de gradiente exponencial (Etest™, bioMérieux, Marcy,l´Etoile, France) en agar *Brucella* con suplemento, depositado en jarras de anaerobiosis con generador de GasPack EZ Pouch™ (Becton Dickinson).

Los antibióticos analizados para los anaerobios fueron ampicilinasulbactam (AS), metronidazol (MZ), clindamicina (CLI), piperacilinatazobactam (TZP), ertapenem (ETP) y meropenem (MEM). Se emplearon los puntos de corte estipulados por el *Clinical and Laboratory Standards Institute* (CLSI) ^8^ y se emplearon las cepas de *Bacteroides fragilis* ATCC 25285 y *Bacteroides thetaiotaomicron* ATCC 2974, como control de calidad.

### Análisis estadístico

Se empleó el programa estadístico Statsdirect™, versión 3.1, para estimar el tamaño de la muestra; se tuvieron en cuenta una positividad esperada de 48 % de los cultivos ^9^, una desviación del 7 % y un intervalo de confianza del 95 %.

Las variables cualitativas se describieron por medio de frecuencias absolutas y relativas, y las variables cuantitativas, por medio de medias y desviaciones estándar; para comparar las proporciones, se usó la prueba de ji al cuadrado.

### Consideraciones éticas

Esta investigación se clasificó como sin riesgo según la Resolución 8430 de 1993 del Ministerio de Salud de Colombia, y fue aprobada por el Comité de Ética e Investigación con Seres Humanos de la Fundación Universitaria de Ciencias de la Salud.

## Resultados

Se analizaron 154 pacientes, 83 (54 % ) hombres y 71 (46 %) mujeres. La media de la edad fue de 29,3 años (desviación estándar, DE= 13,0), con un rango entre 16 y 84 años.

La distribución según la fase de la apendicitis fue: edematosa, 24 % (n=37); fibrinopurulenta, 30 % (n=46) (consideradas como no complicadas); gangrenosa, 22 %, (n=34) y perforada, 24 % (n=37) (consideradas como complicadas).

Se obtuvieron cultivos positivos para aerobios, anaerobios o ambos en el 87 % (n=134) de los pacientes y, aerobios y anaerobios simultáneamente, en el 41 % (n=63); el 77 % (n=118) de los cultivos fueron positivos para aerobios y, el 51 % (n=79), para anaerobios. En la mayoría (81 %) (n=109) de los cultivos positivos, se aislaron dos o más bacterias.

Las cepas de bacterias aerobias y anaerobias identificadas según la fase clínica de la apendicitis, se presentan en los cuadros 1 y 2. La proporción de cultivos positivos fue inferior en la apendicitis no complicada, en comparación con la complicada (80 % (66/83) *Vs*. 95 % (67/71); p=0,003). Las bacterias aerobias y anaerobias aisladas con mayor frecuencia fueron *Escherichia coli* (50 %) (n=79) y *Bacteroides* spp. (25 %) (n=25). La especie de *Bacteroides* más identificada fue *B. thetaiotaomicron*. De los cultivos aerobios, la proporción de aquellos con bacterias Gram negativas fue superior a la de aquellos con Gram positivas (60 % (n=94) Vs. 40 % (n=63); p=0,0002). Los microorganismos aerobios *Staphylococci* coagulasa negativa fueron los segundos en frecuencia y se aislaron en mayor proporción en los casos de apendicitis no complicada que en los de apendicitis complicada (24 % (n=20) Vs. 9,8 % (n=7); p=0,02). En la mitad de los cultivos en que se aisló una bacteria coagulasa negativa, se identificó un segundo microorganismo. En todos los aislamientos con *Enterococcus faecalis*, se aisló un segundo microorganismo y, en el 88 % de ellos, se trataba de *E. coli*.

**Cuadro 1:** Distribución de aislamientos de aerobios según la fase clínica de la apendicitis

	Edematosa	Fibrino- purulenta	Gangrenosa	Perforada	Total		
	n	n	n	n	n	%
						
*Escherichia coli*	11	19	18	31	79	50
*Staphylococcus coagulasa* negativo	9	11	4	3	27	17
*Enterococcus faecalis*	0	2	2	4	8	5
*Staphylococcus aureus* (SARM)	1	0	6	1	8	5
*Streptococcus anginosus*	0	1	0	5	6	4
*Streptococcus* Grupo *Viridans*	2	1	1	1	5	3
*Enterococcus faecium*	2	0	1	2	5	3
*Escherichia coli* (BLEE)	1	2	0	2	5	3
*Kluyvera ascorbata*	1	0	0	1	2	1
*Pseudomonas aeruginosa*	0	0	1	1	2	1
*Vibrio* sp.	0	2	0	0	2	1
*Acinetobacter Iwoffi*	0	1	0	0	1	1
*Aeromonas hidrophyla*	0	1	0	0	1	1
*Enterobacter aerogenes*	0	0	0	1	1	1
*Enterobacter intermedius*	0	1	0	0	1	1
*Enterococcus avium*	1	0	0	0	1	1
*Enterococcus rafinossus*	0	0	0	1	1	1
*Klebsiella pneumoniae*	0	1	0	0	1	1
*Streptococcus agalactiae*	0	1	0	0	1	1
Total	28	43	33	53	157	100

**Cuadro 2:** Distribución de aislamientos de anaerobios según la fase clínica de la apendicitis

	Edematosa	Fibrino-purulenta	Gangrenosa	Perforada	Total		
	n	n	n	n	n	%
						
*Bacteroides* spp.	0	8	3	14	25	25
*Propionibacterium acnes*	4	7	8	2	21	21
*Fusobacterium* spp.	1	4	1	5	11	11
*Clostridium* spp.	3	2	1	3	9	9
*Capnocytophaga* sp.	1	5	0	1	7	7
*Eubacterium* sp.	0	2	2	2	6	6
*Parabacteroides distasonis*		2	1	4	7	7
*Lactobacillus* sp.	2	0	1	2	5	5
*Bifidobacterium dentium*	1	0	1	1	3	3
*Prevotella melaninogenica*	0	0	1	2	3	3
*Porphyromona asaccharolytica*	0	0	0	3	3	3
*Peptostreptococcus magnus*	1	0	0	0	1	1
*Veillonella parvula*	0	0	0	1	1	1
Total	13	30	19	40	102	100

En los estudios de histopatología, se diagnosticaron siete casos de apendicitis grave, en seis de los cuales se identificó *E. coli*; ninguno de estos casos se asoció con bacterias coagulasa negativas.

Todas (n=79) las cepas de *E. coli* aisladas fueron sensibles a carbapenémicos, piperacilina-tazobactam, ceftriaxone, cefepime y amikacina; el 94 % (n=74), a gentamicina, el 90 % (n=71), a ciprofloxacina, y el 30% (n=24), a ampicilina sulbactam. El 6 % (n=5) de las cepas de *E. coli* encontradas eran productoras ß-lactamasas de espectro extendido (BLEE).

El 50 % (n=4) de las cepas de *Staphylococcus aureus* fueron resistentes a la meticilina con 75% de sensibilidad a la clindamicina y el 75 % fueron sensibles a la clindamicina .

En todos los cultivos de anaerobios, la proporción de sensibilidad se distribuyó así: a meropenem, 100 % (n=97); a metronidazol, 10 % (n=10); a ertapenem, 96 % (n=93); a piperacilina-tazobactam, 99 % (n=96); a ampicilina-sulbactam, 93 % (n=90), y a clindamicina, 90 % (n=87). Se detectó resistencia al ertapenem en cepas pertenecientes al género *Fusobacterium*.

Todas las especies (n=33) (100 %) de *Bacteroides* spp. fueron sensibles a metronidazol, piperacilina-tazobactam, ertapenem y meropenem, y el 91% (n=30), a clindamicina y ampicilina-sulbactam.

En total, se presentaron 7 reingresos, de los cuales 5 correspondieron a infección superficial del sitio operatorio y 1 a infección de órgano o espacio del sitio operatorio y, por último, hubo un caso de evisceración que requirió tratamiento quirúrgico.

## Discusión

En Colombia, se han llevado a cabo muy pocas investigaciones para evaluar las bacterias identificadas en la peritonitis secundaria, a saber: el estudio para vigilar las tendencias de la resistencia bacteriana, SMART (*Study for Monitoring Antimicrobial Resistance Trends*) ^9^, estudio multicéntrico internacional patrocinado por la industria farmacéutica; el estudio prospectivo en infección intraabdominal complicada realizado por Vallejo, *et al*. ^5^, y el estudio retrospectivo sobre peritonitis secundaria de Díaz, *et al*. ^6^. En estos estudios, se evaluaron únicamente aislamientos de aerobios. Hasta donde sabemos, este es el primer estudio realizado en Latinoamérica en el que se analizan los aislamientos de anaerobios y su sensibilidad en los casos de apendicitis.

Se eligió el líquido periapendicular como muestra para el cultivo ya que, en este fluido, se pueden identificarlos microorganismos con capacidad de 'traslocar' la mucosa intestinal y que podrían estar más implicados en la patogénesis del proceso inflamatorio. Las muestras der la luz intestinal podrían estar más correlacionadas con el microbioma intestinal.

La proporción de cultivos positivos (87 %) (n=134) es superior a la descrita por otros investigadores, quienes reportan cifras en un rango de 18 a 98 % ^6^^,^^10^^-^^15^. Estas variaciones pueden obedecer a los criterios de inclusión empleados, como el tipo de apendicitis, el medio de transporte, las demoras en la siembra y las técnicas de recuperación. El alto porcentaje de recuperación en este estudio puede obedecer, en parte, a la siembra inmediata en las salas de cirugía y a la inoculación directa en los botellas de hemocultivos. En el 81 % (n=109) de los cultivos positivos, se identificó más de un microorganismo, porcentaje superior al 60 % descrito por Guillet-Caruba, *et al*. ^12^, y al 18 % encontrado por Jeon, *et al*. ^13^. Las proporciones de cultivos positivos encontradas en los casos de apendicitis no complicada (80 %, 66/83) y de la complicada (95 %, 67/71), son superiores al 24,3 % y al 59 %, respectivamente, reportadas en el estudio español de García-Marín, *et al*. ^16^.

En este estudio, la bacteria aerobia aislada con mayor frecuencia (53 %) (n=84) fue *E. coli*; este hallazgo es constante en todos los estudios sobre infección intrabdominal, con una frecuencia que oscila entre 66,7 % y 81 % ^12^^,^^13^^,^^17^. El 6 % (5/84) de las cepas de *E. coli* fueron productoras de BLEE, cifra que está dentro del rango publicado de 3,5 % a 16,3 % ^5^^,^^10^^,^^13^. La resistencia encontrada a ampicilina-sulbactam para esta enterobacteria fue de 70 %, porcentaje superior al informado por García-Marín, *et al*. ^16^,(2,4 %), al de Vallejo, *et al*., en Colombia en pacientes con infección intrabdominal de cualquier origen, (34 %)^5^, al de la red de vigilancia multicéntrica en Colombia de *E. coli* de cualquier origen, (39 %) ^3^ y al del SMART, (58,6 %). La resistencia a ciprofloxacina fue de tan solo 10 % (n=8), frecuencia inferior a la del SMART (30 %), lo que podría explicarse por la restricción que existe a nivel nacional del uso intrahospitalario de la ciprofloxacina como parte de los programas de optimización de antimicrobianos.

Entre las bacterias aerobias, *Staphylococci* coagulasa negativa ocupan el segundo lugar en frecuencia; hacen parte de la microbiota intestinal y, si bien podrían considerarse contaminantes de los cultivos, su papel en la patógenesis de la infección intraabdominal se ha evidenciado en los últimos años y, además, se ha demostrado su capacidad de inducir necrosis de coagulación en el intestino ^18^. Llama la atención que el número de aislamientos de estas bacterias es mayor en las fases de apendicitis no complicada, lo cual puede estar reflejando el papel preponderante de bacterias más virulentas en la fases complicadas. No hay estudios previos que reporten esta bacteria en los casos de apendicitis.

Dejando de lado las cepas coagulasa negativas, los Gram positivos más frecuentes fueron *Streptococci* (12 %) y *Enterococci* (6,3 %), resultados concordantes con los obtenidos por otros investigadores ^14^^,^^15^. El hallazgo, aunque en un bajo porcentaje (4 % de los aerobios), del grupo *anginosus* de *Streptococcus* ratifica el papel de este agente patógeno en la generación de abscesos en diferentes localizaciones; Boueil, *et al*., en su estudio de Nueva Caledonia, lo consideran el segundo microorganismo en frecuencia, solo superado por *E. coli*^19^.

A pesar de que la virulencia de *Enterococci* está establecida, su baja incidencia en la infección intraabdominal condujó a que la *Infectious Diseases Society of America* recomendara su cubrimiento antibiótico empírico únicamente en las infecciones intraabdominal intrahospitalarias, en pacientes críticamente enfermos, en pacientes con válvulas protésicas y en adultos mayores ^7^. En los casos de apendicitis, esto justificaría el empleo de algunos antibióticos con poca afinidad por este microorganismo, como carbapenémicos, aminoglucósidos diferentes de gentamicina, cefalosporinas, clindamicina y metronidazol.

Solo en dos casos (1%) se aisló *Pseudomonas aeruginosa*, en estudios previos, la frecuencia de esta bacteria es muy variable, en el estudio multicéntrico de Coccolini su frecuencia fue también baja (3,3%) ^17^.Sin embargo, otros autores de diferentes latitudes ubican a este microorganismo en un segundo lugar entre los Gram negativos, con proporciones entre el 10 y el 16 % ^9^^,^^15^^,^^16^^,^^19^.

No se encontraron factores de riesgo, como antecedentes de hospitalizaciones o prescripción de antibióticos, en los pacientes en los cuales se aisló S. aureus resistente a la meticilina o cepas de *E. coli* productoras de BLEE, lo cual podría sugerir un cambio en la microbiota intestinal de la comunidad, debido a la exposición a alimentos contaminados con estas cepas.

El porcentaje de recuperación de anaerobios (51 %) (n=79) es muy superior a lo reportado, con un rango entre 4,2 % y 20 % ^11^^,^^12^^,^^14^^,^^15^. En esta serie, el anaerobio más aislado fue *Bacteroides* spp., lo cual concuerda con todos los estudios; no obstante, el hallazgo de B. thetaiotaomicron como la especie más frecuente, difiere de lo reportado en otras latitudes. La frecuencia de *Propionibacterium acnes* (21 %, n=21)es superior a la notificado y, aunque muchas cepas exhiben resistencia al metronidazol, en esta muestra todos los aislamientos fueron sensibles.

La resistencia a la clindamicina en 9 % (3/33) de las cepas aisladas de *Bacteroides* spp. es menor a la descrita por otros investigadores, con porcentajes del 10 % al 40 % ^5^; en un estudio español, se encontró 30,8 % de resistencia ^16^. De forma similar, la resistencia a ampicilina-sulbactam de 9 % (n=3/33), es inferior al 26 % reportado por la Red de Vigilancia Microbiológica de la Comunidad Valenciana ^20^.

Este estudio tiene como limitante que fue realizado en un solo centro y que no se contó con técnicas de biología molecular o de espectrometría de masas que permitirían mayor precisión en la determinación de especies.

A pesar de que la práctica de tomar muestras para cultivo en casos de apendicitis cayó en desuso en las dos últimas décadas por considerarse que los microorganismos aislados eran usualmente los mismos; en el momento actual de emergencia de bacterias multirresistentes, esta práctica retoma su pertinencia con el fin de establecer el patrón epidemiológico y el protocolo de antibioticoterapia regional, particularmente, en los casos de apendicitis complicada.

Debido a la resistencia de *E. coli* y de *Bacteroides* spp. a la ampicilinasulbactam, no se recomienda su empleo en el tratamiento de la infección intraabdominal. En los esquemas terapéuticos que incluyen varios antibióticos, se debe preferir el metronidazol sobre la clindamicina como anaerobicida y se deben llevar a cabo estudios epidemiológicos locales para establecer la frecuencia de *P. aeruginosa*, con el fin de de incluir a este no fermentador en los protocolos antibióticos dirigidos.

En los centros donde no se cuente con medios de transporte apropiados, la inoculación directa del líquido periapendicular en botellas de hemocultivos en las salas de cirugía, permite una alta recuperación de bacterias aerobias y anaerobias.
